# Staphylococcus aureus Prophage-Encoded Protein Causes Abortive Infection and Provides Population Immunity against Kayviruses

**DOI:** 10.1128/mbio.02490-22

**Published:** 2023-02-13

**Authors:** Lucie Kuntová, Ivana Mašlaňová, Radka Obořilová, Hana Šimečková, Adéla Finstrlová, Pavol Bárdy, Marta Šiborová, Liudmyla Troianovska, Tibor Botka, Petr Gintar, Ondrej Šedo, Zdeněk Farka, Jiří Doškař, Roman Pantůček

**Affiliations:** a Department of Experimental Biology, Faculty of Science, Masaryk University, Brno, Czech Republic; b Central European Institute of Technology, Masaryk University, Brno, Czech Republic; c Department of Biochemistry, Faculty of Science, Masaryk University, Brno, Czech Republic; d Department of Chemistry, York Structural Biology Laboratory, University of York, York, United Kingdom; e National Centre for Biomolecular Research, Faculty of Science, Masaryk University, Brno, Czech Republic; University of Tennessee at Knoxville

**Keywords:** *Staphylococcus aureus*, lysogeny, phage resistance, abortive infection, *Kayvirus*, cell death, phage therapy, bacteriophage evolution, bacteriophage therapy, bacteriophages

## Abstract

Both temperate and obligately lytic phages have crucial roles in the biology of staphylococci. While superinfection exclusion among closely related temperate phages is a well-characterized phenomenon, the interactions between temperate and lytic phages in staphylococci are not understood. Here, we present a resistance mechanism toward lytic phages of the genus *Kayvirus*, mediated by the membrane-anchored protein designated Pdp_Sau_ encoded by Staphylococcus aureus prophages, mostly of the Sa2 integrase type. The prophage accessory gene *pdp*_Sau_ is strongly linked to the lytic genes for holin and ami2-type amidase and typically replaces genes for the toxin Panton-Valentine leukocidin (PVL). The predicted Pdp_Sau_ protein structure shows the presence of a membrane-binding α-helix in its N-terminal part and a cytoplasmic positively charged C terminus. We demonstrated that the mechanism of action of Pdp_Sau_ does not prevent the infecting kayvirus from adsorbing onto the host cell and delivering its genome into the cell, but phage DNA replication is halted. Changes in the cell membrane polarity and permeability were observed from 10 min after the infection, which led to prophage-activated cell death. Furthermore, we describe a mechanism of overcoming this resistance in a host-range *Kayvirus* mutant, which was selected on an S. aureus strain harboring prophage 53 encoding Pdp_Sau_, and in which a chimeric gene product emerged via adaptive laboratory evolution. This first case of staphylococcal interfamily phage-phage competition is analogous to some other abortive infection defense systems and to systems based on membrane-destructive proteins.

## INTRODUCTION

Bacteriophages are widespread across ecosystems and are among the most numerous entities on earth. Bacteria are constantly threatened by phage infection, and 20 to 40% of daily bacterial death is caused by bacteriophages (1). Conversely, the integrated genomes of prophages often promote beneficial phenotypic changes in their hosts (2) and play a major role in horizontal gene transfer, thus contributing to the evolution of bacterial pathogens (3).

Most Staphylococcus aureus isolates carry multiple siphoviral prophages in their genome (4) with an impact on virulence, toxin production, immune evasion, and host preference (5) as well as the mobilization of variable genetic elements (6) and dissemination of antibiotic resistance by transduction (7–9). Besides temperate staphylococcal siphoviruses, there are the strictly lytic myoviruses (10) and podoviruses (11) that are believed to be suitable for use as antimicrobial agents.

Over the last 2 decades, interest in using phages for the treatment of bacterial infections has increased enormously. Based on current clinical trials, phage therapy is efficient, safe, and acceptable for treatment in human medicine (12). The therapeutic potential of kayviruses with the type phage K (13) led to the characterization of many phage strains of this genus with an extremely broad host range (14) as was demonstrated for phage 812 (15). Kayviruses were studied to clarify their polyvalence (15, 16), lytic activity (17–19), synergistic effect with antibiotics (20), and interaction with the immune system (21); to describe their structure and genome delivery (22); and for safety assessments for therapy (23, 24). This progress in the implementation of kayviruses into safe phage therapy has been achieved by the characterization of staphylococcal phage-host interactions at the omics level, such as comparative genomics (10, 14, 15, 25), transcriptomics (26, 27), and proteomics studies (28).

One of the limitations of using kayviruses in medical practice is the possible resistance of staphylococcal species to these phages. During the constant competition between phages and their hosts, multiple bacterial phage resistance systems targeting various stages of the phage life cycle have evolved (29, 30). In staphylococci, these include various mechanisms, such as targeting foreign DNA by restriction modification (31), CRISPR-Cas immunity (32), protection against entry into the host cell through the modification of wall teichoic acids (33), overproduction of staphylococcal protein A (34), prophage-induced immunity (35), interference with phage reproduction mediated by pathogenicity islands (36), and the different host factors required for phage reproduction (37).

Some antiphage systems in staphylococci are yet to be discovered, namely, the large group of mechanisms categorized as abortive infections. Here, we report that temperate siphoviruses can protect the staphylococcal population from destruction by virulent *Kayvirus* phages by an abortive infection mechanism.

## RESULTS AND DISCUSSION

### Prophages induce insensitivity of S. aureus to kayviruses.

Prophageless S. aureus strains ISP8 and 1039 were lysogenized by prophages 11, 29, 42E, 47, 53, 71, 77, 80α, 83A, 84, 85, or 96 and tested for susceptibility to lytic kayviruses 812, 812a, and K. The integration of prophages 47, 53, and 80α resulted in a resistant phenotype to the lytic phages 812 and K. Phage 53 was used for the lysogenization of multiple strains, including methicillin-resistant S. aureus (MRSA) USA300 (Table 1), which led to the establishment of a resistant phenotype to phages 812 and K independently of the genetic background of the lysogens. In models of cured strains NCTC 8511 (53^+^)c and ISP8 (53^+^)c, it was shown that sensitivity to both phages 812 and K is renewed after the prophage is lost. Similarly, S. aureus NCTC 8325, which is naturally resistant to phage 812, regained its sensitivity after all of its prophages were removed (Table 1). In contrast to phages 812 and K, prophages 47, 53, and 80α do not affect sensitivity to phage 812a, the phage 812 host-range mutant that originated during passaging phage 812 on S. aureus strain NCTC 8511 with prophage 53 (38) (Table 1).

**TABLE 1:** Staphylococcal strains used in this study and their sensitivity to lytic phages^*a*^

Bacteria and strain	Presence of *pdp*_Sau_	Prophage content; strain characteristics	Reference or source	Sensitivity to kayvirus		
				K	812	812a
S. aureus						
RN4220	−	No prophage; propagation strain for phage K	95	+	+	+
CCM 4028 (= SA812)	−	Sa2int, Sa3int; propagation strain for phage 812	38	+	+	+
RN4220 (pCN51)	−	No prophage	This study	+	+	+
RN4220 (pCN51-*pdp*_Sau_)	+	No prophage	This study	−	−	+
RN4220 (pCN51-*pdp*_Sau_-orf812a_191)	+	No prophage	This study	+	+	+
RN4220 (53^+^)	+	Sa7int	This study	−	−	+
RN4220 (53^+^) (pCN51-orf812a_191)	+	Sa7int	This study	+	+	+
NCTC 8325	+	Sa2int, Sa3int, Sa5int	Culture collection	−	−	+
ISP8 (= 8325-4)	−	No prophage; cured for phi11, phi12, phi13	96	+	+	+
ISP8 (47^+^)	+	Sa2int	This study	−	−	+
ISP8 (53^+^)	+	Sa7int	64	−	−	+
ISP8 (53^+^)c	−	No prophage; cured for phi53	64	+	+	+
1039 (= CCM 4890)	−	No prophage	97	+	+	+
NCTC 8511	−	Sa2int, Sa3int, Sa6int	Culture collection	+	+	+
NCTC 8511 (53^+^) (= S26)	+	Sa2int, Sa3int, Sa6int, Sa7int	38	−	−	+
NCTC 8511 (53^+^)c	−	Sa2int, Sa3int, Sa6int, cured for phi53	This study	+	+	+
USA300	−	Sa2int, Sa3int	98	+	+	+
USA300c	−	No prophage; cured for phiSa2_USA_ and Sa3_USA_	99	+	+	+
USA300c (53^+^)	+	Sa7int	This study	−	−	+
E48 (= NRL 02/947)	+	Sa2int, Sa3int, clinical MRSA isolate	100	−	−	+
S. epidermidis						
Tü 3298	−	ND	101	+	+	−
Tü 3298 (pCN51-*pdp*_Sau_)	+	ND	This study	−	−	−

a +, sensitive; −, resistant; ND, not determined.

The studied prophages 47, 53, and 80α that induced immunity to kayviruses 812 and K are unrelated or only distantly related to each other based on their whole-genome sequence. Phage 47 belongs to group A by the original serological classification and to integrase type Sa2. Phages 53 and 80α belong to group B and integrase types Sa7 and Sa5, respectively. Each integrase type possesses a different *att* site; thus, the insertion inactivation of a bacterial gene is not the cause of the induced nonsensitivity. Comparative genomic analyses of phages 47, 53, and 80α revealed only one common gene corresponding to ORF016 coding for an unknown protein (UniProtKB accession no. Q4ZDJ8) in the previously published bacteriophage 53 genome (39). This gene was designated *pdp*_Sau_ (phage defense protein of S. aureus).

A set of MRSA strains was subsequently screened for the *pdp*_Sau_ gene using PCR. All *pdp*_Sau_-positive strains exhibited resistance to wild-type phage 812. Strain E48 (ST8/t024/staphylococcal cassette chromosome *mec* element IV [SCC*mec* IV]) related to USA300 (40) as a representative of the most frequent genotype that naturally carried the *pdp*_Sau_ gene (Table 1) was genome sequenced and used for further analyses.

### Phage sensitivity assay in an artificial expression system.

**FIG 1:**
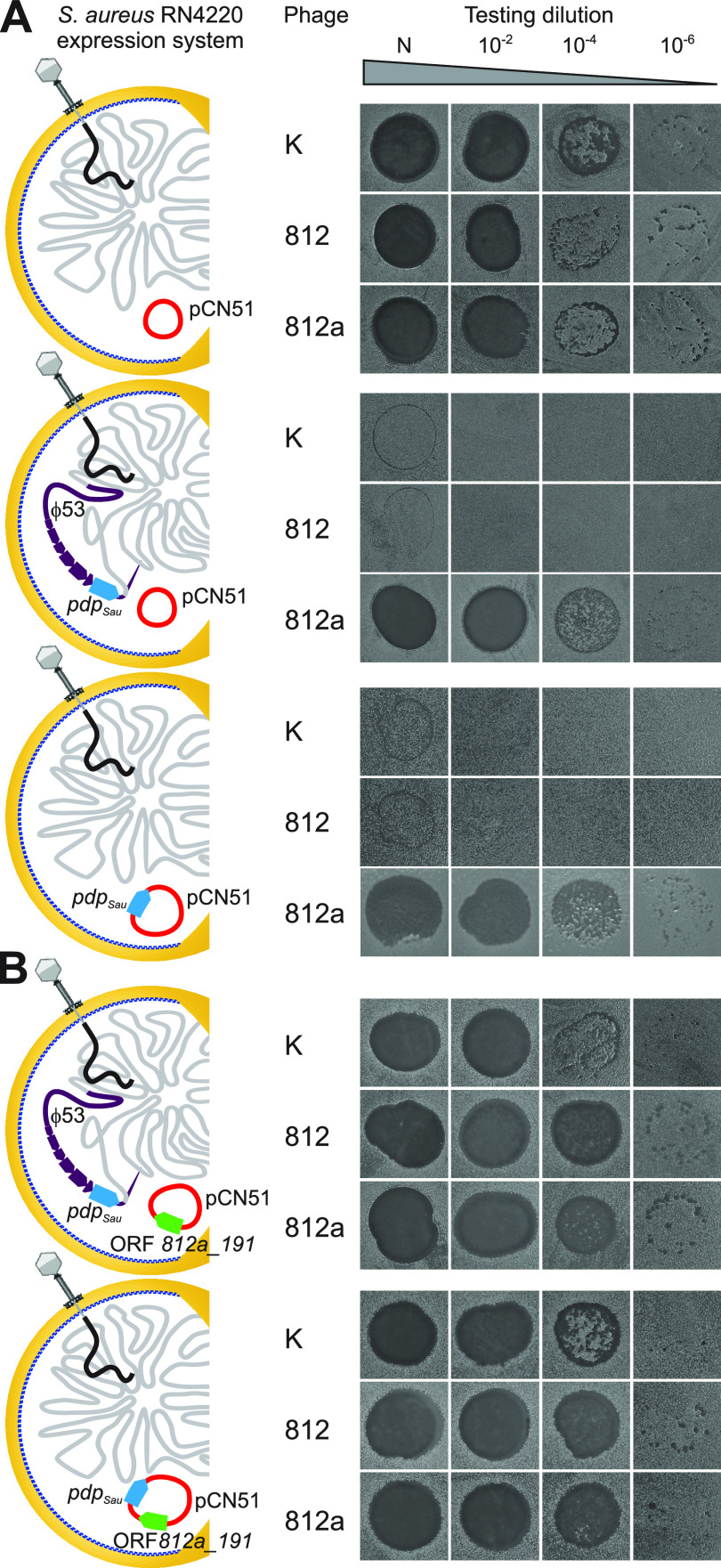
Impact of *pdp*_Sau_ gene expression on *Kayvirus* lytic action demonstrated by drop assay performed with phages K, 812, and 812a on S. aureus RN4220 derivatives. Testing was performed at four phage concentrations, nondiluted phage with a titer of 10^9^ PFU/mL (N) and dilutions of 10^−2^, 10^−4^, and 10^−6^. Four types of resulting lytic zones were distinguished—confluent lysis, semiconfluent lysis, single plaques, growth inhibition, or no lysis. If single plaques appeared at any dilution, the strain was considered susceptible. (A) Phage-sensitive control strain RN4220 (pCN51) compared to wild-type phage resistant RN4220 (53^+^) (pCN51) and RN4220 (pCN51-*pdp*_Sau_) harboring *pdp_Sau_* gene on prophage 53 or plasmid pCN51, respectively. Phage 812a replicates effectively on strains with the *pdp*_Sau_ gene. (B) S. aureus strains RN4220 (53^+^) (pCN51-ORF812a_191) and RN4220 (pCN51-*pdp*_Sau_-ORF812a_191) coexpressing *pdp*_Sau_ and ORF 812a_191, which restores the sensitive phenotype. The restoration of the phage-sensitive phenotype occurs both in the lysogenic strain with prophage-encoded *pdp_Sau_* and the strain with coexpressed *pdp_Sau_* and ORF 812a_191 from a common promoter.

To verify the direct association between the *pdp*_Sau_ gene and a phage-resistant phenotype, the *pdp*_Sau_ gene was cloned in the expression vector pCN51 under a cadmium-inducible promoter (41) and electroporated into S. aureus RN4220, which is naturally sensitive to phages 812 and K. The Pdp_Sau_ protein was detected by mass spectrometry after SDS-PAGE of the proteins from lysed cells both in the naturally occurring lysogen and the artificial system, where 28% of the protein sequence (amino acid range 39 to 178) was covered by nine tryptic peptides. A strain harboring the construct pCN51-*pdp*_Sau_ exhibited a resistant phenotype to both phages after the overexpression, the same as the lysogenic strain S. aureus RN4220 (53^+^) (Fig. 1A). This proved that the protein Pdp_Sau_ alone is sufficient to induce resistance to phages 812 and K. Next, the construct pCN51-*pdp*_Sau_ was transformed into the coagulase-negative Staphylococcus epidermidis Tü 3298 strain (Table 1), where the expression of the *pdp*_Sau_ gene also led to the induction of a resistant phenotype.

### The *pdp*_Sau_ gene prevalence in S. aureus whole-genome sequences.

**FIG 2:**
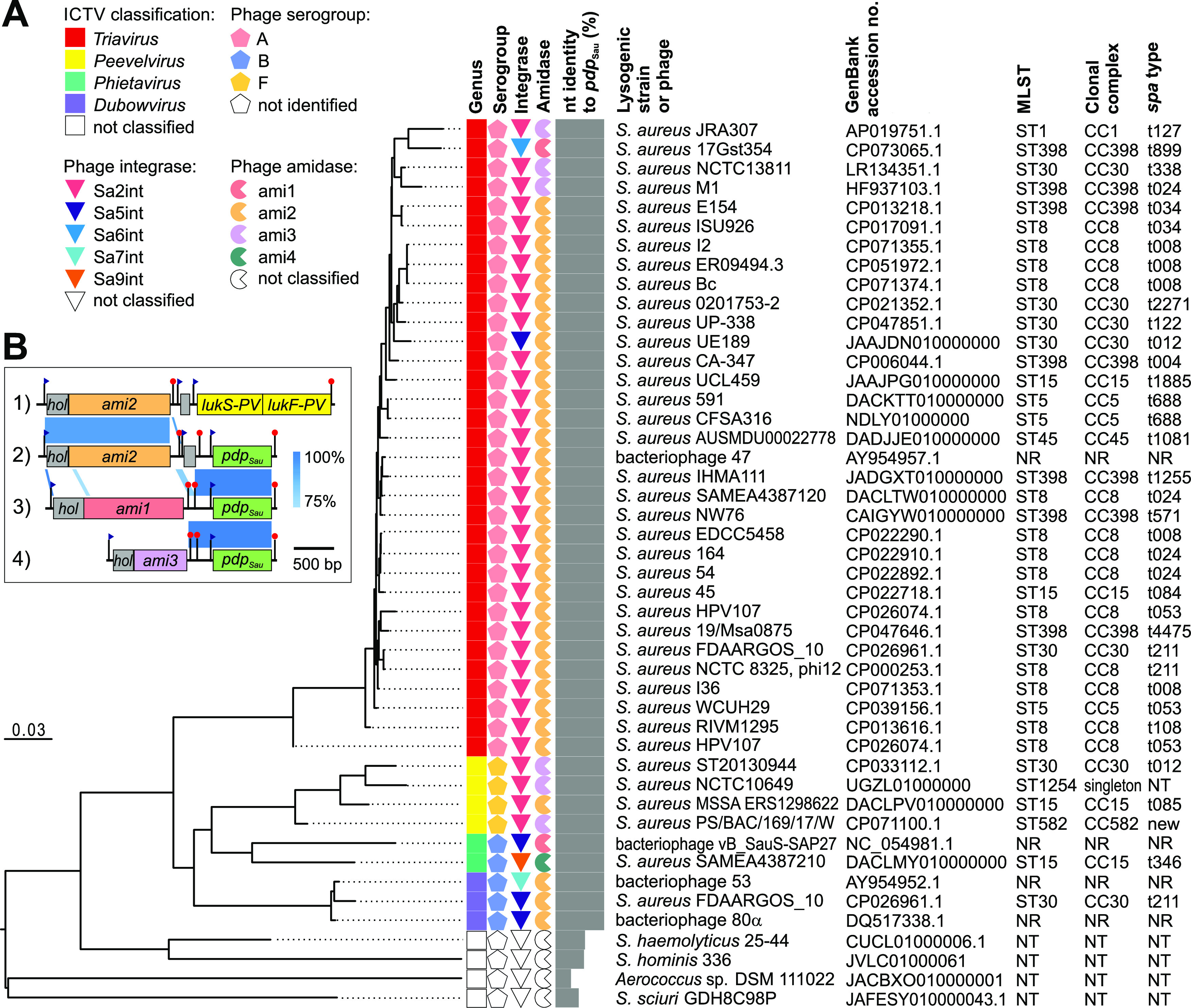
Phylogenetic insights into prophages harboring *pdp*_Sau_ gene identified in whole-genome sequences of *Staphylococcaceae* family and their characteristics. (A) Bacteriophage and prophage genomes extracted from whole-genome sequences that matched *pdp*_Sau_ were compared using the Genome-BLAST Distance Phylogeny method with the settings recommended for prokaryotic viruses (90). The resulting intergenomic distances were used to infer a balanced minimum evolution tree with branch support via FASTME including Subtree Pruning and Regrafting (SPR) postprocessing (91). The branch lengths of the resulting tree are scaled in terms of the respective distance formula used. Phage genomes were characterized by phage type corresponding to serological group (65), integrase gene type (44), and amidase gene type (42). The nucleotide identity of *pdp*_Sau_ homologs to ORF016 of phage 53 is shown. Multilocus sequence type and staphylococcal protein A (*spa*) type were derived from the genome assemblies. NR, not relevant; NT, not typeable. (B) Nucleotide sequence alignment showing the gene structure of lytic modules and accessory genes in the genomes of four S. aureus prophages as follows: (i) phi PVL (92), (ii) phi 53 (39), (iii) prophage vB_StaphS-IVBph354 (93), and (iv) prophage from strain PS/BAC/169/17/W (94). ORFs with proven or predicted functions are depicted as colored boxes. Nucleotide identity between genomic regions is indicated by blue-shaded regions. Putative promoters and terminators are depicted as blue flags and red pins, respectively.

Analyses of the prevalence of *pdp*_Sau_-encoding prophages in more than 60 thousand *Staphylococcaceae* genomes available in the NCBI database matched a set of 41 bacterial genomes with this gene. The *pdp*_Sau_ gene was found solely on S. aureus prophage regions. A gene with lower similarity was found in the prophage genomes of coagulase-negative staphylococci and the genus *Aerococcus* (Fig. 2A). The extracted prophage sequences were classified by BLAST search and *in silico* PCR into the genera *Triavirus*, *Phietavirus*, *Peevelvirus*, and/or *Dubowvirus* (Fig. 2A). The most frequent ones (85%) were prophages harboring Sa2 integrase, and the rest comprised integrase types Sa5, Sa6, Sa7, and Sa9. The gene organization of the lytic module shows that *pdp*_Sau_ is always localized downstream of the gene for amidase in the lysis module (Fig. 2B) and is typically linked to amidase ami2 according to the previous classification system (42). The presence of the *pdp*_Sau_ gene adjacent to ami2 was also confirmed by PCR in multiple lysogenic MRSA strain E48. The ami2 lytic module of *pdp*_Sau_-positive phages was homologous with Panton-Valentine leukocidin (PVL)-converting phages (100% nucleotide [nt] identity) (Fig. 2B), which leads to the hypothesis that it has the same origin as in PVL-encoding phages (43). As determined previously (44), the crossover point for the integration of the PVL toxin-encoding complex is situated at the end of the phage amidase open reading frame (ORF), and the *pdp_Sau_* locus possibly recombines at this crossover point.

Next, we tested whether there was a relationship between the presence of the *pdp*_Sau_ gene and clonal complexes of whole-genome sequenced lysogenic strains. Predominant genotypes from clonal complexes CC5, CC8, CC15, CC30, and ST398 (Fig. 2A) are typical for community isolates where prophage-induced immunity to kayviruses may represent an evolutionary advantage, e.g., for survival and dissemination in a wastewater environment as an important reservoir for lytic phages (45).

### Transmembrane protein Pdp_Sau_ is structurally similar to apoptotic protein.

**FIG 3:**
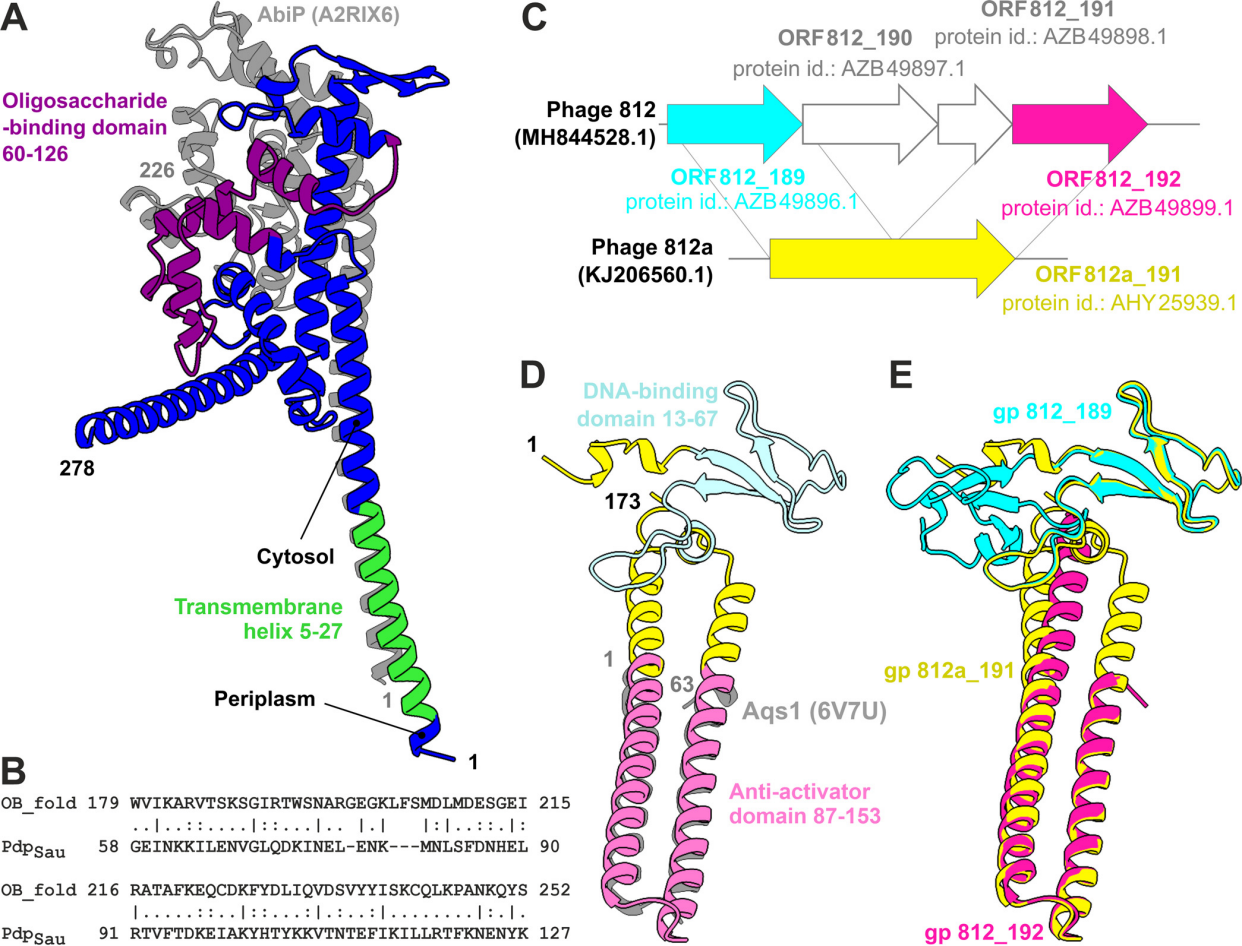
Structure prediction of immunity protein Pdp_Sau_ and new fusion protein restoring phage sensitivity encoded by phage mutant 812a. (A) 3D structure prediction of Pdp_Sau_ (blue) with N-terminal transmembrane helix (lime green) and oligosaccharide-binding fold-like region (purple) highlighted. The structure is superimposed with the predicted structure of characterized immunity protein AbiP (A2RIX6) from *Lactococcus* (gray). (B) EMBOSS Needle pairwise alignment (BLOSUM62) of oligosaccharide-binding (OB) domains of RPA replication protein (UniProtKB accession no. Q24492) with OB_fold hit in Pdp_Sau_ protein (score, 25; similarity, 40.5%). (C) Sequence alignment of genomic loci encoding ORF 812_189 and ORF 812_192 in phage 812 and fusion ORF 812a_191 in the genome of host-range mutant phage 812a, depicting the emerging deletion. (D) 3D structure prediction of fusion protein ORF812a_191, superimposed with the solved structure of the antiactivator Aqs1 (PDB accession no. 6V7U; gray). (E) Structural alignment of gene products encoded by ORF 812_189 (cyan), ORF 812_192 (magenta), and fusion ORF 812a_191 (yellow).

Region 5 to 27 of the 278-amino acid (aa) protein Pdp_Sau_ contains many hydrophobic residues and protein topology prediction classified it as a transmembrane domain (TMHMM, probability of N-in, 0.985) (Fig. 3A). Furthermore, structure comparison of the Pdp_Sau_ protein region 3 to 28 suggests a weak similarity of the region to Enterococcus faecalis RNAI protein (2KV5) and S. aureus PepA1 protein (4B19) (see Table S1 in the supplemental material). RNAI forms part of the *par* locus and has the function of an Fst toxin for the stable maintenance of plasmids in cells (46). Fst translocated across a membrane induces changes in membrane integrity, leading to the disruption of cell division (47). PepA1 protein, similarly to Fst, forms a membrane-binding α-helix in a 22-residue part of its N terminus (48). The liquid chromatography-tandem mass spectrometry (LC-MS/MS) analysis (see Table S2 in the supplemental material) revealed a predominant allocation of Pdp_Sau_ protein in the membrane fraction compared to the cytoplasmic one; therefore, we assume Pdp_Sau_ is embedded in the membrane. The cytoplasmic C-terminal domain of Pdp_Sau_ is positively charged. Pairwise sequence alignment (Fig. 3B) confirmed the presence of an oligosaccharide-binding fold (UniProtKB accession no. Q24492), which often facilitates DNA-binding function (49). Both transmembrane and DNA-binding domains were described in the lactococcal phage-defense abortive system protein AbiP (UniProtKB accession no. A2RIX6) (50). We thus compared protein models generated by Alphafold2 of Pdp_Sau_ to AbiP, which despite the low amino acid identity exhibited a significant structural similarity demonstrated by their pairwise superimposing (Fig. 3A; see also Table S1).

10.1128/mbio.02490-22.5 TABLE S1 Calculated similarities of predicted Pdp_Sau_ protein and its domain structures. Download Table S1, XLSX file, 0.01 MB. Copyright © 2023 Kuntová et al. 2023 Kuntová et al. https://creativecommons.org/licenses/by/4.0/ This content is distributed under the terms of the Creative Commons Attribution 4.0 International license.

10.1128/mbio.02490-22.6 TABLE S2 Proteins identified from membrane and cytoplasmic fractions by LC-MS/MS analysis. Download Table S2, XLSX file, 2.6 MB. Copyright © 2023 Kuntová et al. 2023 Kuntová et al. https://creativecommons.org/licenses/by/4.0/ This content is distributed under the terms of the Creative Commons Attribution 4.0 International license.

Based on protein structure predictions, we hypothesize that Pdp_Sau_ is one of the apoptotic-like membrane proteins associated with abortive systems (Abi), whose modes of action involve degradation or depolarization of the cell membrane (50). In Gram-positive bacteria, Abi systems provide resistance against phage infection that can lead to cell death and target various stages of the cell life cycle (50–52). Other classes of Abi systems target tRNAs or mRNAs and cleave essential proteins in the host translation apparatus, resulting in growth arrest and nonproductive infection (53), or compete with native replicase proteins (54).

### The *pdp*_Sau_ gene does not affect *Kayvirus* adsorption and genome release but blocks its DNA replication.

To study the mechanism of action of Pdp_Sau_, the strains RN4220, RN4220 (53^+^), and RN4220 (pCN51-*pdp*_Sau_) were infected with the wild-type phage 812. The reaction of strains to infection with phage was monitored by turbidity assay. Strains expressing *pdp*_Sau_ exhibited growth of the bacterial culture upon infection with phage 812, which was slowed down for the first 4 h compared to uninfected culture, whereas the absence of the *pdp*_Sau_ gene led to lysis of the culture (see Fig. S1 in the supplemental material). The expression level of the *pdp*_Sau_ gene during phage 812 infection was determined using quantitative reverse transcription-PCR (RT-qPCR). Neither overexpression nor downregulation of the *pdp*_Sau_ gene was detected at 0, 5, 10, and 20 min of infection with phage 812 compared to the sample without the addition of phage (see Table S3 in the supplemental material). The change in the expression of the *pdp*_Sau_ gene is therefore not essential for the activation of the phage defense mechanism.

10.1128/mbio.02490-22.1 FIG S1 Growth characteristics of S. aureus strains expressing *pdp*_Sau_ gene during infection with phages 812 or 812a determined using a densitometric assay. Download FIG S1, PDF file, 1.4 MB. Copyright © 2023 Kuntová et al. 2023 Kuntová et al. https://creativecommons.org/licenses/by/4.0/ This content is distributed under the terms of the Creative Commons Attribution 4.0 International license.

10.1128/mbio.02490-22.7 TABLE S3 Quantification of *pdp*_Sau_ gene expression in Staphylococcus aureus RN4220 (53^+^) during phage infection. Download Table S3, XLSX file, 0.02 MB. Copyright © 2023 Kuntová et al. 2023 Kuntová et al. https://creativecommons.org/licenses/by/4.0/ This content is distributed under the terms of the Creative Commons Attribution 4.0 International license.

Next, we examined specific steps of the phage life cycle—adsorption, genome delivery, transcription, and replication—to determine at which point the cycle is arrested. The comparison of adsorption rate (see Fig. S2 in the supplemental material) showed that phage 812 virions adsorb efficiently onto both *pdp*_Sau_-positive and -negative cells; thus, Pdp_Sau_ does not affect adsorption.

10.1128/mbio.02490-22.2 FIG S2 Adsorption kinetics of bacteriophages 812 and 812a on S. aureus strains expressing the *pdp*_Sau_ gene. Download FIG S2, PDF file, 1.3 MB. Copyright © 2023 Kuntová et al. 2023 Kuntová et al. https://creativecommons.org/licenses/by/4.0/ This content is distributed under the terms of the Creative Commons Attribution 4.0 International license.

We recently showed that *Kayvirus* transcription starts immediately after the entry of phage DNA into the host cells (26). To verify that *Kayvirus* phage 812 genome delivery and the transcription of its DNA occurs in *pdp*_Sau_-positive S. aureus strains, the transcripts of the early (anti-sigma factor, orf 812_132; GenBank accession no. AZB49840.1), middle (putative DNA-binding protein, 812_143; GenBank accession no. AZB49851.1), and late (baseplate wedge protein, 812_118; GenBank accession no. AZB49826.1) phase of infection (26) were quantified by RT-qPCR (see Fig. S3 in the supplemental material). The presence of transcripts of all of the tested loci confirmed that the DNA of phage 812 is inside cells and accessible to the host transcription apparatus. Based on the facts that phage DNA is present inside the cell and Pdp_Sau_ is structurally similar to lactococcal AbiP, we hypothesize that similarly to AbiP, the binding of a nucleic acid to Pdp_Sau_ activates the phage resistance mechanism.

10.1128/mbio.02490-22.3 FIG S3 Relative quantification of phage 812 transcripts in S. aureus RN4220 (53^+^) using RT-qPCR. Download FIG S3, PDF file, 1.3 MB. Copyright © 2023 Kuntová et al. 2023 Kuntová et al. https://creativecommons.org/licenses/by/4.0/ This content is distributed under the terms of the Creative Commons Attribution 4.0 International license.

The replication of phage 812 was examined using the absolute quantification of the *mcp* gene for major capsid protein during infection (see Fig. S4 in the supplemental material). Rees and Fry in their original study of the phage K replisome (55) described that during the first half of the latent period, the number of phage DNA molecules increased from 1 copy to 27 phage equivalents. This observation is consistent with quantitative PCR (qPCR) results, where we detected about a 30-fold increase in the amount of phage 812 DNA in the sensitive control strain RN4220 20 min postinfection (Fig. S4). After 30 min, the copy number of the *mcp* gene increased about 40-fold in control RN4220 compared to those of *pdp*_Sau_-positive strains, where the *mcp* copy number only increased 2-fold. The very low increase in the genome copy number leads to the presumption that the phage replication in *pdp*_Sau_-positive strains is either not activated or is stalled at the very beginning.

10.1128/mbio.02490-22.4 FIG S4 Absolute quantification of major capsid gene (*mcp*) using qPCR to indicate that phage DNA replication has stopped during 812 phage infection in S. aureus strains expressing the *pdp*_Sau_ compared to S. aureus RN4220 (pCN51). Download FIG S4, PDF file, 1.3 MB. Copyright © 2023 Kuntová et al. 2023 Kuntová et al. https://creativecommons.org/licenses/by/4.0/ This content is distributed under the terms of the Creative Commons Attribution 4.0 International license.

### *pdp*_Sau_ impacts cell membrane potential and permeability in *Kayvirus*-infected cells.

**FIG 4:**
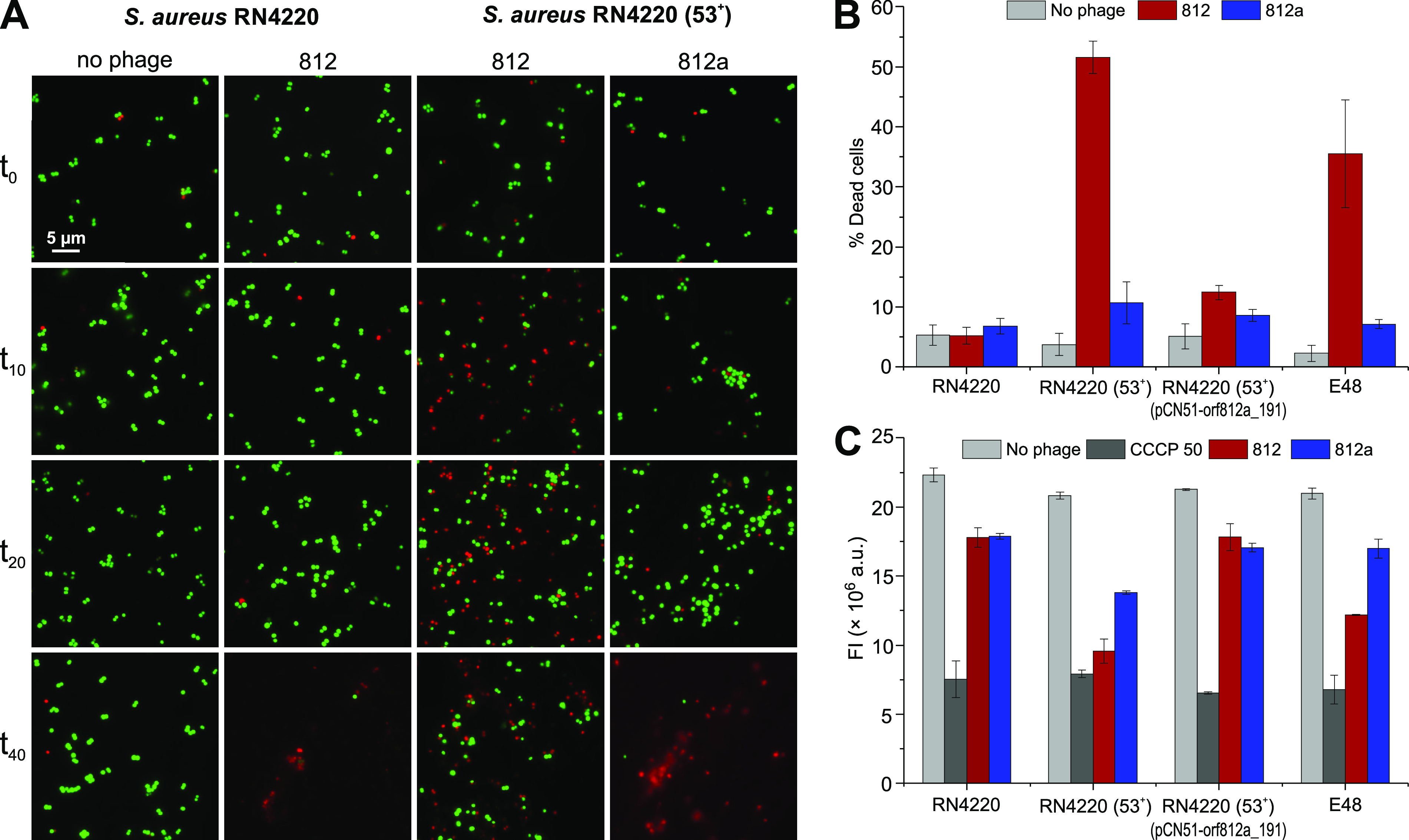
Changes in S. aureus membrane permeability through Pdp_Sau_-based phage defense mechanism illustrated by LIVE/DEAD staining. (A) Fluorescence microscopy of S. aureus infected with phages 812 or 812a and no phage control sample, visualized by fluorescence microscopy at time points 0, 10, 20, and 40 min after the onset of infection. Propidium iodide, a red-fluorescent nucleic acid-binding dye, which cannot enter cells with intact membranes, was used as a marker for the loss of membrane integrity and cell death. Dead cells are represented by red dots and live cells by green dots. (B) Percentage of dead bacterial cells 10 min after the onset of infection. S. aureus cultures were infected with phages 812 or 812a and compared with noninfected strains. (C) Changes in membrane polarity examined by BacLight bacterial membrane potential kit assay on S. aureus RN4220, RN4220 (53^+^), RN4220 (53^+^) (pCN51-orf812a_191), and clinical MRSA strain E48 treated with phages 812 or 812a at an MOI_input_ of 10. Treatment with the ionophore carbonyl cyanide 3-chlorophenylhydrazone, component B (CCCP) at a final concentration of 50 mM was used as a positive control of depolarization. The decrease in membrane potential was measured as a loss of red fluorescence emitted by carbocyanine dye DiOC_2_(3) (3,3′-diethyloxa-carbocyanine iodide).

Due to the assumed transmembrane localization of Pdp_Sau_ protein, the changes in the cell membrane integrity 10 to 40 min postinfection in strains RN4220 and RN4220 (53^+^) were assessed using LIVE/DEAD cell staining. In the RN4220 strain, we observed live cells until the release of new phage progeny after 40 min (Fig. 4A). Compared to the *pdp*_Sau_ gene-negative strain RN4220, we observed a presence of dead cells 10 min postinfection and subsequent increase in cell counts with no cell lysis after 40 min postinfection in *pdp*_Sau_ gene-positive strain RN4220 (53^+^) (Fig. 4A and B). This indicates halted phage propagation followed by a rapid growth of live cells starting from 20 min after infection (Fig. 4A). In this way, the bacterial population survives due to an abortive defense mechanism.

Changes in membrane permeability are connected with membrane potential. The carbocyanine dye staining showed a statistically significant reduction in red fluorescence (*P* value < 0.01) in the *pdp*_Sau_-negative nonlysogenic strain RN4220 compared to that in the *pdp*_Sau_-positive lysogenic strain RN4220 (53^+^), indicating a change in membrane potential (Fig. 4C). This was also observed in the control MRSA strain E48 (Fig. 4B and C). Similarly, the abortive Rex system of bacteriophage lambda characterized by termination of macromolecular synthesis, loss of active transport, ATP hydrolysis, and altruistic cell death is explained by depolarization of the cytoplasmic membrane due to activation of the membrane component of the system (56).

### Kayviruses can escape prophage-induced bacterial resistance mechanism.

Bacteriophage 812 host-range mutants are capable of growing on *pdp*_Sau_-positive strains. This capability was first observed in a mutant designated 812a, which was obtained as rare plaques after plating phage 812 (mutation frequency, 9.9 × 10^−9^; efficiency of plating, 0.43) on the lysogenic strain S. aureus NCTC 8511 (53^+^) (38). Phage 812a propagated efficiently on all of the analyzed lysogenic *pdp*_Sau_-positive strains, as determined by phage drop plaque assays (Table 1; Fig. 1A), turbidity assay (Fig. S1), and microscopic observation of the infected cells (Fig. 4A).

Whole-genome sequencing of phage 812a (15) revealed a deletion leading to a new fusion gene with a possible role in overcoming the action of Pdp_Sau_. The deletion in phage 812 genome includes a locus encoding four hypothetical genes (ORF 812_189 to ORF 812_192; GenBank accession no. MH844528.1). After the deletion, ORF 812_189 and ORF 812_192 form a fusion gene in phage 812a annotated as ORF 812a_191 (GenBank accession no. KJ206560.1) (Fig. 3C and E). The new 173-aa fusion protein has a conserved DNA-binding domain on its N terminus (residues 13 to 67) with a zinc finger motif similar to Miz-1 protein (HHpred, 2N26; probability, 98.81%; e value, 6.4e−9) (57) and to the transcription repressor CTCF from eukaryotes (HHpred, 6QNX, probability: 98.4%, e-value 1.4e−7) (58). The tertiary structure of the protein in the C-terminal region 87 to 153 is similar to the antiactivator Aqs1 of *Pseudomonas* phage DSM3 (DALI search, PDB accession no. 6V7U; Z-score = 6.5) (Fig. 3D), which is involved in blocking a host phage-resistance mechanism by inhibiting the DNA-binding domain of a host cell regulator (59).

To verify the direct association of the new fusion gene with overcoming the resistance mechanism, ORF 812a_191 was coexpressed with *pdp*_Sau_ under one promoter in S. aureus RN4220 (pCN51-*pdp*_Sau_-orf812a_191), which led to restored sensitivity to phages 812 and K. The same phenomenon was confirmed in the S. aureus RN4220 (53^+^) (pCN51-orf812a_191) system, which naturally carries *pdp*_Sau_ in the genome (Fig. 1B; see also Fig. S1). No change in membrane potential or permeability was observed when S. aureus strain RN4220 (53^+^) (pCN51-orf812a_191) was infected with phage 812 (Fig. 4B and C).

### Conclusions.

The described defense mechanism encoded by a prophage accessory gene protects the staphylococcal bacterial population against virulent lytic phages via abortive infection. Because it is encoded by prophages in various clonal lineages, we assume it was spread by horizontal gene transfer. An analogy can be found in the abortive mechanisms of lactococci (53) but even in a group of Gram-negative bacteria (60), where the responsible genes are also carried by mobile genetic elements. The interaction of the prophage gene product with the infecting phage halts the replication of its DNA and leads to changes in the permeability of the cell membrane. Based on these findings, we conclude that the infected part of the bacterial host population is sacrificed to stop the lytic infection by the *Kayvirus* and prevent the release of its new progeny. The bacteria benefit from a lysogenic conversion that allows them to escape the lytic action of *Kayvirus* at the population level. Therefore, we believe that this novel mechanism of phage competition in staphylococci leads to the stable maintenance of prophages protecting their hosts. Kayviruses can evolve through mutations and regain the ability to lyse lysogenic strains, thus maintaining their wide range of hosts, which is important for their use in phage therapy.

## MATERIALS AND METHODS

### Bacterial and bacteriophage strains and culture conditions.

The strains used in this study are listed in Table 1. Staphylococcal strains were routinely grown in meat peptone broth (MPB) and/or meat peptone agar (MPA) according to Botka et al. (15). Escherichia coli strains were grown at 37°C with shaking at 160 rpm in LB medium. Phage 812, deposited in the Czech Collection of Microorganisms under number CCM 7911, and phage 812a were described previously (38). Bacteriophage K was kindly provided by G. Xia (University of Manchester, UK) (61). S. aureus phages from the International Typing Set (62) and phage 80α (63) were described previously. Lysogenized strains were prepared as previously described (64), and the presence of prophages was verified by PCR (65). The phage-cured S. aureus strain USA300 (designated USA300c) was generated by deleting native prophages Sa2int_USA300_ and Sa3int_USA300_ using the plasmid pKOR1 as described for S. aureus Newman (66). Phage-cured strains ISP8 (53^+^)c and NCTC 8511 (53^+^)c were prepared by using UV light and recognized by replica plating on MPA medium inoculated with an indicator strain (64).

### Construction of plasmid vectors and protein preparation.

The expression vectors constructed in this study are derived from high-copy-number shuttle vector plasmid pCN51 with cadmium-inducible promoter (41) and are listed in Table 1. The protein-coding regions were amplified by PCR with primers designed for restriction enzyme cloning (see Table S4 in the supplemental material). Restriction endonucleases BamHI and EcoRI (New England Biolabs) were used for cloning by ligation with T4 DNA ligase (New England Biolabs). Plasmid constructs were transformed into competent E. coli Top10F′ (Invitrogen) and then into E. coli BL21(DE3) (Invitrogen) for protein expression or transferred into electrocompetent S. aureus cells (67). All constructs were verified by Sanger sequencing in the Eurofins MWG Operon sequencing facility (Ebersberg, Germany). The expression and coexpression of cloned genes from plasmid constructs were verified by mass spectrometry.

10.1128/mbio.02490-22.8 TABLE S4 Lists of oligonucleotides designed in this study. Download Table S4, XLSX file, 0.01 MB. Copyright © 2023 Kuntová et al. 2023 Kuntová et al. https://creativecommons.org/licenses/by/4.0/ This content is distributed under the terms of the Creative Commons Attribution 4.0 International license.

### Phage susceptibility testing.

The double agar overlay technique (MPA with 2 mM CaCl_2_) was used for phage susceptibility testing and the isolation of phage mutants. The phage lysates of a titer of 10^9^ PFU/mL were diluted up to 10^−6^ and applied in triplicates by spotting 10-μL aliquots onto soft agar lawns inoculated with the tested S. aureus strain. Plates were incubated overnight at 37°C. The strain was only evaluated as sensitive if the phage formed plaques.

### Adsorption assays.

The adsorption efficiency of phages 812 and 812a onto S. aureus strains RN4220 and RN4220 (53^+^) was determined as described previously (33). Briefly, the adsorption was analyzed using a multiplicity of infection (MOI_input_) of 0.1, and the adsorption rate (%) was calculated by determining the number of unbound phage particles in the supernatant and subtracting it from the total number of input PFU as a ratio of the total number of input PFU. The adsorption rate was estimated 5 min after phage infection.

### Bacterial cell growth assays during phage infection.

Bacterial strains were cultivated aerobically in 20 mL of MPB to the logarithmic phase (optical density at 600 nm [OD_600_] = 0.4 to 0.45) at 37°C. A transparent 96-well cell culture plate (SPL Life Sciences) with a transparent cover and an Infinite 200 PRO (Tecan) microplate reader were used for the turbidimetric assay. The experiments were carried out in triplicates in a total volume of 200 μL per well at 37°C with continuous orbital shaking (amplitude, 4 mm) for 24 h using the recommended protocol and instrument settings (absorbance, 600 nm; 20 flashes; 0 ms settle time). Phage infection assay was done at an MOI_input_ of 5 or 10 with the addition of CaCl_2_ to a final concentration of 2 mM.

### DNA extraction for phage gene quantification in infected cells.

Bacterial culture grown to an OD_600_ of 0.4 to 0.45 in 50 mL MPB at 37°C was mixed with phages 812 or 812a at an MOI_input_ of 0.1 and incubated with shaking. The 1.5-mL aliquots were taken at sampling time points 0, 2, 5, 10, 15, 20, and 30 min and centrifuged at 10,000 × *g* for 2 min. Pellets were frozen using liquid nitrogen and kept at −80°C. DNA was extracted from each aliquot sample using a High Pure PCR template preparation kit (Roche) with prolonged lysis with lysostaphin (Sigma-Aldrich) added to a final concentration of 10 μg/mL.

### cDNA preparation.

Total RNA was extracted using the TRI reagent (Sigma-Aldrich) from S. aureus cells infected with phages at sampling time points 0, 2, 5, 10, 15, 20, and 30 min, harvested as described above. The procedure was done in RNase-free tubes according to the manufacturer’s instructions with the following modifications for the lysis of Gram-positive bacteria: 10^8^ cells were lysed in 1 mL of TRI reagent and transferred to lysing matrix B with 0.1 mm silica spheres (MP Biomedicals) and homogenized for 2 min. The silica spheres were collected by centrifugation for 3 min at 10,000 × *g* at 4°C. Purified RNA was used for cDNA synthesis in a reverse transcription assay using a high-capacity cDNA reverse transcription kit (Applied Biosystems).

### qPCR and RT-qPCR of phage genes at different times of infection.

Each reaction mixture (20 μL) contained 10 μL of 2× LightCycler 480 SYBR green I master (Roche), forward and reverse primers (each 10 μM) listed in Table S4, and template DNA or cDNA diluted into a volume of 5 μL. Reactions were carried out in triplicates using a LightCycler 480 Instrument II (Roche) according to Mašlaňová et al. (68). An initial denaturation of DNA at 95°C for 10 min was followed by 30 cycles of amplification (95°C for 15 s, 55°C for 20 s, 72°C for 15 s) and a dissociation phase at 95°C for 15 s, 60°C for 60 s, 95°C for 5 s, and 60°C for 15 s. The amplification efficiency of qPCR was calculated from threshold cycle (*C_T_*) values of standard curves prepared from the plasmid or genomic DNA for each reaction, and a linear regression curve through the data points was generated. The measurements were done in biological and technical triplicates. The expression level was analyzed from crossing point (Cp) values using a one-way analysis of variance (ANOVA) test. All statistical analyses were performed in R v4.2.1. (https://cran.r-project.org/).

### Protein identification by mass spectrometry.

Vertical one-dimensional SDS-PAGE was performed as described previously (69). Separation zones corresponding to the molecular weight of the expected protein (33 ± 5 kDa) were excised from the gel, and after destaining and washing procedures, they were digested with trypsin (Promega) for 2 h at 40°C. Tryptic peptides extracted from gels were subjected to LC-MS/MS analysis using an UltiMate 3000 RSLCnano liquid chromatography system (Thermo Fisher Scientific) connected on-line to an Impact II ultra-high resolution Qq-time-of-flight mass spectrometer (Bruker, Germany). MS/MS data were searched against a custom database of expected amino acid sequences and in parallel against the NCBIprot database (https://ftp.ncbi.nih.gov/blast/db/FASTA/) using an in-house Mascot search engine version 2.4.1 (Matrix Science, UK).

To obtain cytoplasmic (C) and membrane (M) protein fractions, the isolation method described previously (70) was used with changes for the final processing of the membrane fraction. The pelleted membrane fraction was washed twice with 50 mM ammonium bicarbonate (AB), centrifuged at 20,000 × *g* for 10 min, and solubilized in SDT lysis buffer (4% SDS, 0.1 M dithiothreitol, 0.1 M Tris-HCl, pH 7.6). Solubilized proteins were processed using filter-aided sample preparation (FASP) and digested with SOLu-trypsin dimethylated (Merck) in 50 mM AB. Recovered peptides were cleaned using ethyl acetate extraction. LC-MS/MS analyses of both fractions were performed in an RSLCnano liquid chromatography system on-line connected to an Orbitrap Exploris 480 mass spectrometer (Thermo Fisher Scientific). Peptides were separated using an analytical EASY-Spray column (Acclaim PepMap C_18_ column; 2-μm particles, 75 μm × 500 mm; Thermo Fisher Scientifics; part number ES903) during a 138-min gradient elution (mobile phase A, 0.1% formic acid in water; mobile phase B, 0.1% formic acid in 80% acetonitrile). MS data were acquired in a data-dependent strategy with a defined number of scans based on precursor abundance with survey scan (*m/z* 350 to 2,000). The resolution of the survey scan was 120,000 (at *m/z* 200) with a target value of 1 × 10^6^ ions and maximum injection time of 500 ms. High-energy collisional dissociation-tandem mass spectrometry (HCD-MS/MS) data (30% relative fragmentation energy) were recorded at 15,000 resolution (maximum injection time, 50 ms). MaxQuant software version 2.0.3.0 with inbuild search engine Andromeda (Max-Planck-Institute of Biochemistry) was used for data evaluation. Searches were done against the S. aureus NCTC 8325 reference proteome (UP000008816) and cRAP contaminants database version 2012.01.01. Carbamidomethylation of cysteine was set as a fixed modification while oxidation (M), deamidation (N, Q), and acetylation (protein N-term) were set as variable modifications. Trypsin was used as the protein-cleaving enzyme with two allowed missed cleavages. Peptides and proteins with a false discovery rate of <1% were considered for final data evaluation. All identified proteins including the protein of interest (UniProtKB accession no. Q2FYE0) are listed in Table S2 in the supplemental material. Mass spectrometry data were deposited in the ProteomeXchange Consortium via the PRIDE partner repository under database identifier PXD036676.

### Bacterial membrane permeability and membrane polarity assays.

S. aureus strains RN4220, RN4220 (53^+^), RN4220 (53^+^) (pCN51-orf812a_191), and E48 were routinely grown in MPB. The bacterial culture was 100-fold diluted in MPB supplemented with 0.5 μM Cd(NO_3_)_2_ to induce expression where needed and incubated at 37°C with shaking to an OD_600_ of 0.35 to 0.40. The phage 812 or 812a was added at an MOI_input_ of 5. The bacterial cells without phages were used as a negative control. The 1.5-mL aliquots were taken at sampling times 0, 10, 20, and 40 min, centrifuged at 10,000 × *g* for 2 min, and the pellets were washed once and resuspended in 150 μL of 50 mM Tris-HCl (pH 7.5) for membrane permeability assay and in 150 μL of phosphate-buffered saline (PBS) buffer for membrane polarity assay. The cell suspension was stained using a LIVE/DEAD BacLight bacterial viability kit (Invitrogen) as recommended by the manufacturer. The kit contains SYTO 9 and propidium iodide (PI), which have different permeabilities through the bacterial membrane. The stained bacterial samples were observed with an Olympus BX41 fluorescence microscope (Olympus, Japan). The SYTO 9 emission was observed using a fluorescein isothiocyanate (FITC) filter cube (U-MWB2; excitation 475 ± 30 nm; emission >520 nm; dichroic mirror (DM) 500 nm) and the PI using a tetramethyl rhodamine isocyanate (TRITC) filter cube (U-MWG2; excitation 530 ± 40 nm; emission >590 nm; DM 570 nm). The measurements were done in biological and technical triplicates.

Changes in membrane polarity were detected with a BacLight bacterial membrane potential kit (Invitrogen) containing carbocyanine dye DiOC_2_(3) (3,3′-diethyloxa-carbocyanine iodide) and compared with the control ionophore carbonyl cyanide 3-chlorophenylhydrazone, component B (CCCP) at a final concentration of 50 μM, both diluted in dimethyl sulfoxide (DMSO). The precipitation of DiOC_2_(3) indicates changes in potential at the bacterial membrane, and its natural green emission shifts to red (71). The decrease in membrane potential was observed as a loss of red fluorescence using an Upcon S-Pro reader (Labrox, Finland) in a black nonbinding 96-well microplate (Greiner, Austria). Kinetic measurements were performed from the addition of ionophores for 1 h, and the intensity value was evaluated at 20 min. The filters used in this case were 485 ± 10 nm for excitation and 616 ± 8.5 nm for emission (DM excitation, 450 to 492 nm; emission, 520 to 550 nm). The measurements were done in biological and technical triplicates. The effect of phage addition on membrane depolarization in the tested strains was analyzed using analysis of variance (ANOVA) followed by *post hoc* Tukey tests performed in R v4.2.1.

### Protein structure prediction.

The transmembrane domain of Pdp_Sau_ was predicted using TMHMM version 2.0 (72). The DNA-binding domain in Pdp_Sau_ was predicted using DRNApred (73). HHpred (74), Phyre2 (75), and DALI search (76) were used for Pdp_Sau_, estimating the similarity to distantly related proteins based on the secondary and tertiary structure prediction. The three-dimensional (3D) models of Pdp_Sau_ and orf812a_191 protein structures were predicted by AlphaFold, developed by DeepMind (77). Chimera version 1.15.rc (78) and ChimeraX version 1.2.5 (79) were used for the visualization of 3D protein structures.

### Whole-genome sequencing.

The bacterial culture was prepared and enzymatically treated as previously described (80). The genomic DNA was extracted using a Genomic DNA Clean & Concentrator-25 kit (Zymo Research) according to the manufacturer’s instructions. For sequencing on the Oxford Nanopore platform, the library was prepared using an SQK-RAD004 rapid sequencing kit (Oxford Nanopore Technologies) according to the manufacturer’s instructions. The library was sequenced with a FLO-FLG001 flow cell (R9.4.1) in a MinION device (Oxford Nanopore Technologie). The device was controlled with MinION software release 22.05.5 (Oxford Nanopore Technologies). Basecalling, demultiplexing, and barcode trimming were performed using standalone ONT Guppy software version 6.1.7 using the config file dna_r9.4.1_450bps_sup.cfg with a default minimum q-score threshold of 10. For Illumina-based sequencing, the 500-bp sequencing library was prepared with an xGen DNA Lib Prep EZ (Integrated DNA Technologies, Belgium). The samples were sequenced using a 600v3 Miseq sequencing cartridge in a 2 × 300 paired end mode using an Illumina MiSeq sequencing platform (Illumina). Illumina reads were trimmed and filtered using Trimmomatic version 0.38.1 with the sliding window model using average quality required 20 (81). The complete bacterial genome sequence was obtained using a hybrid assembly with Unicycler version 0.4.8 (82) using a minimal k-mer size of 0.2 and highest k-mer size of 0.95 with 10 k-mer steps used in a SPAdes assembly. The resulting assembly was polished with Pilon version 1.24 (83). The genome was annotated using the NCBI Prokaryotic Genome Annotation Pipeline (84).

### Genomic sequences used in this study and bioinformatic analyses.

Whole genomic sequences with the *pdp*_Sau_ gene were identified by BLAST search (https://blast.ncbi.nlm.nih.gov/). Prophage sequences from this data set were extracted manually based on their integration sites (42). The PubMLST website was used for multilocus sequence type (MLST) determination (85). Spa-types were derived with RIDOM Spa server (86). Phage and prophage genomes were characterized based on *in silico* PCR (87) using primers targeting structural genes corresponding to the serological group (65), integrase gene type (44), and amidase genes (42). Promoter sequences were predicted using the BPROM webserver (88), and terminator sites were predicted using ARNold (89).

### Data availability.

The complete genome sequence of S. aureus strain E48 (=NRL 02/947) has been deposited in GenBank/ENA/DDBJ under accession numbers CP103850 (chromosome) and CP103849 (plasmid). The associated BioProject and BioSample accession numbers are PRJNA873286 and SAMN30496472, respectively.
