# Supplementary material for: Staphylococcus aureus Prophage-Encoded Protein Causes Abortive Infection and Provides Population Immunity against Kayviruses
Source: mBio. 2023 Feb 13;14(2):e02490-22. doi: 10.1128/mbio.02490-22 (PMC10127798; doi:10.1128/mbio.02490-22)
Supplement: FIG S1 [file mbio.02490-22-s0005.pdf]

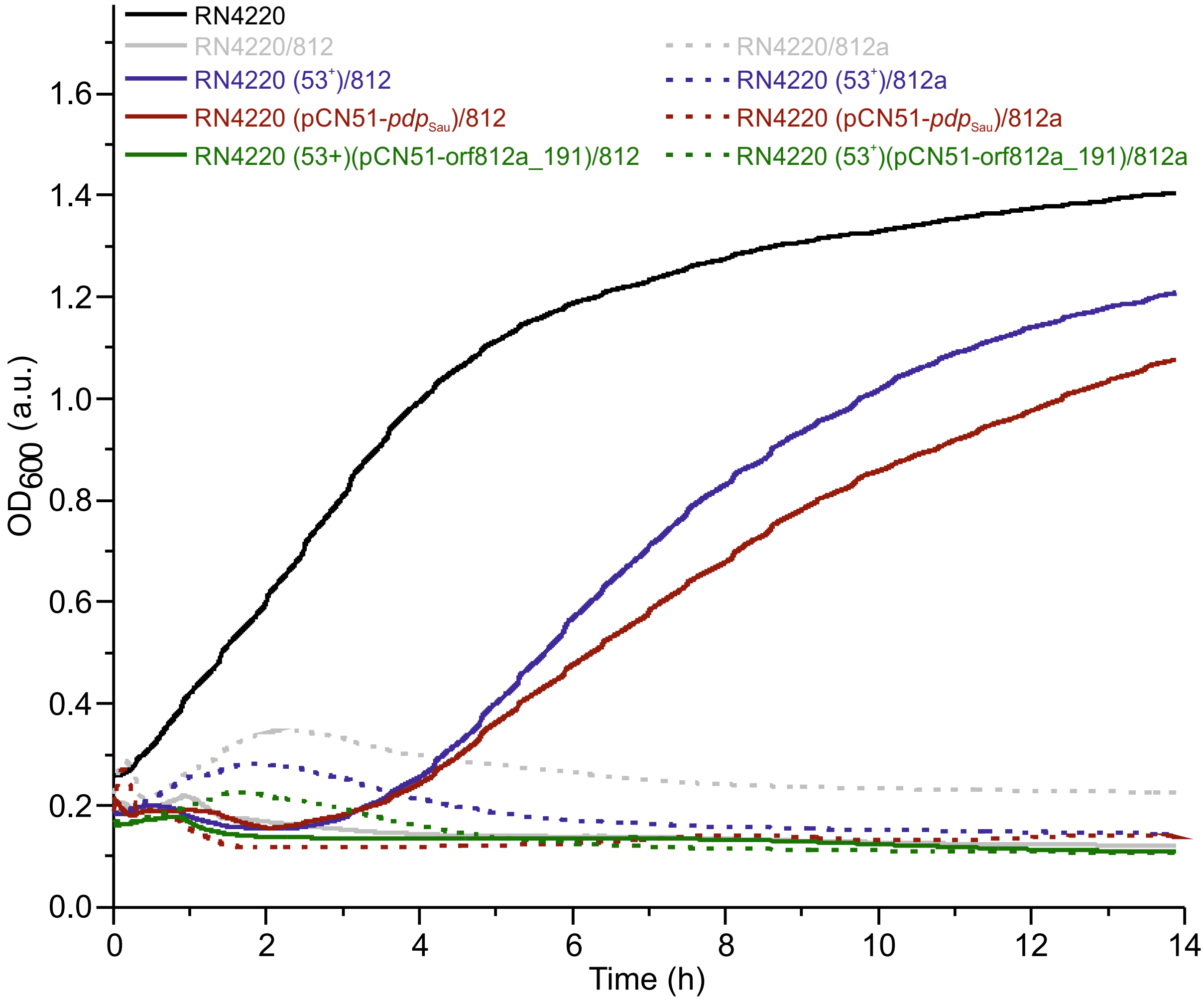

FIG S1 Growth characteristics of *Staphylococcus aureus* strains expressing *pdp*$_{\text{Sau}}$ gene during infection with phage 812 or its host-range mutant 812a determined using a densitometric assay. The growth curve of non-infected strain *S. aureus* RN4220 (in black) was used as a control. Growth curves of infected strains with phage 812 (solid line) or 812a (dashed line) are color-coded according to the legend.
