# Supplementary material for: Staphylococcus aureus Prophage-Encoded Protein Causes Abortive Infection and Provides Population Immunity against Kayviruses
Source: mBio. 2023 Feb 13;14(2):e02490-22. doi: 10.1128/mbio.02490-22 (PMC10127798; doi:10.1128/mbio.02490-22)
Supplement: FIG S2 [file mbio.02490-22-s0006.pdf]

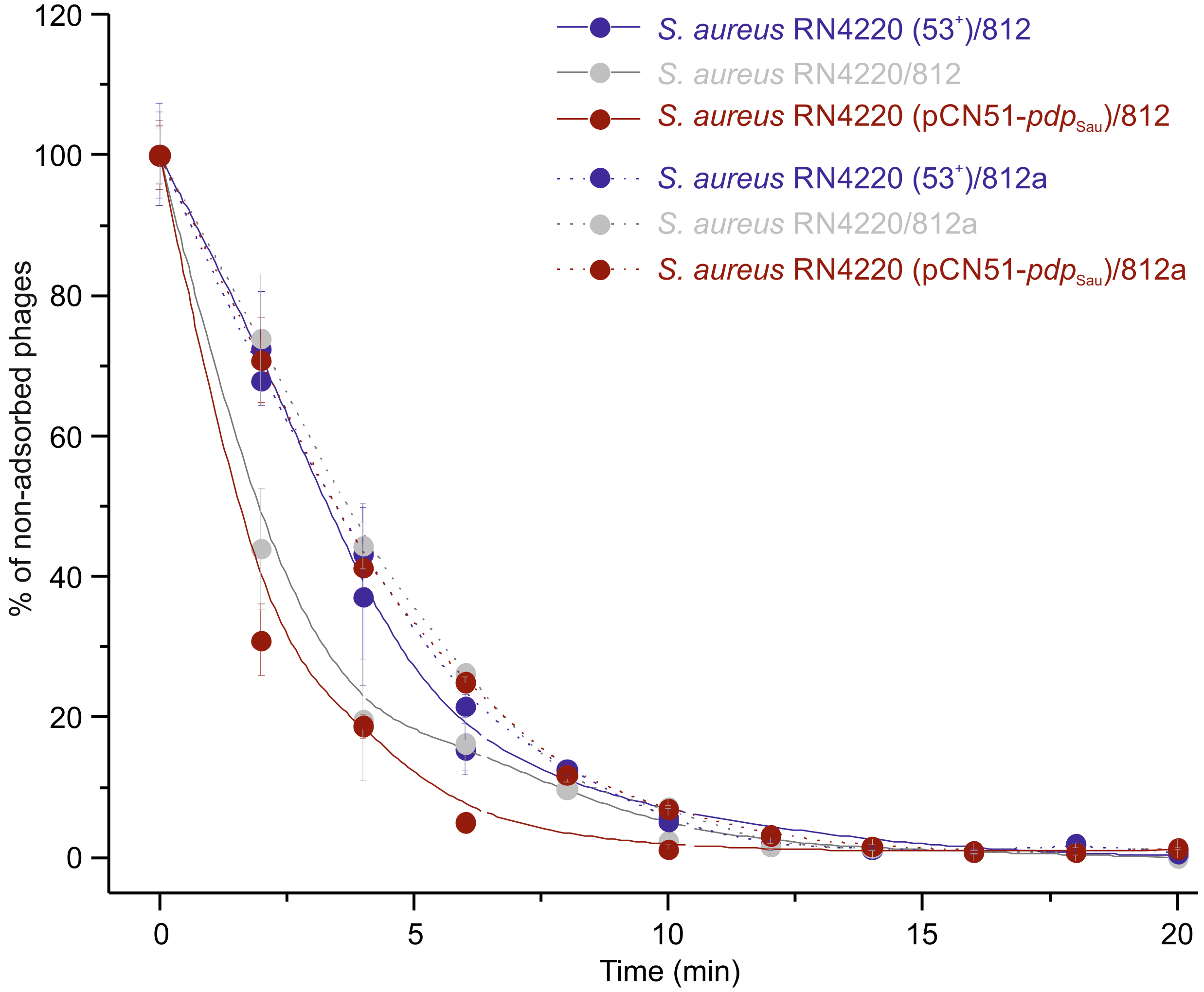

FIG S2 Adsorption kinetics of bacteriophages 812 (solid line) and its host-range mutant 812a (dashed line) on *Staphylococcus aureus* strains expressing the pdp_{Sau} gene. The adsorption rate was calculated by determining the number of unbound phage particles in the supernatant and subtracting it from the total number of input PFU. Analyzed strains are color-coded according to the legend.
