# Supplementary material for: Staphylococcus aureus Prophage-Encoded Protein Causes Abortive Infection and Provides Population Immunity against Kayviruses
Source: mBio. 2023 Feb 13;14(2):e02490-22. doi: 10.1128/mbio.02490-22 (PMC10127798; doi:10.1128/mbio.02490-22)
Supplement: FIG S3 [file mbio.02490-22-s0007.pdf]

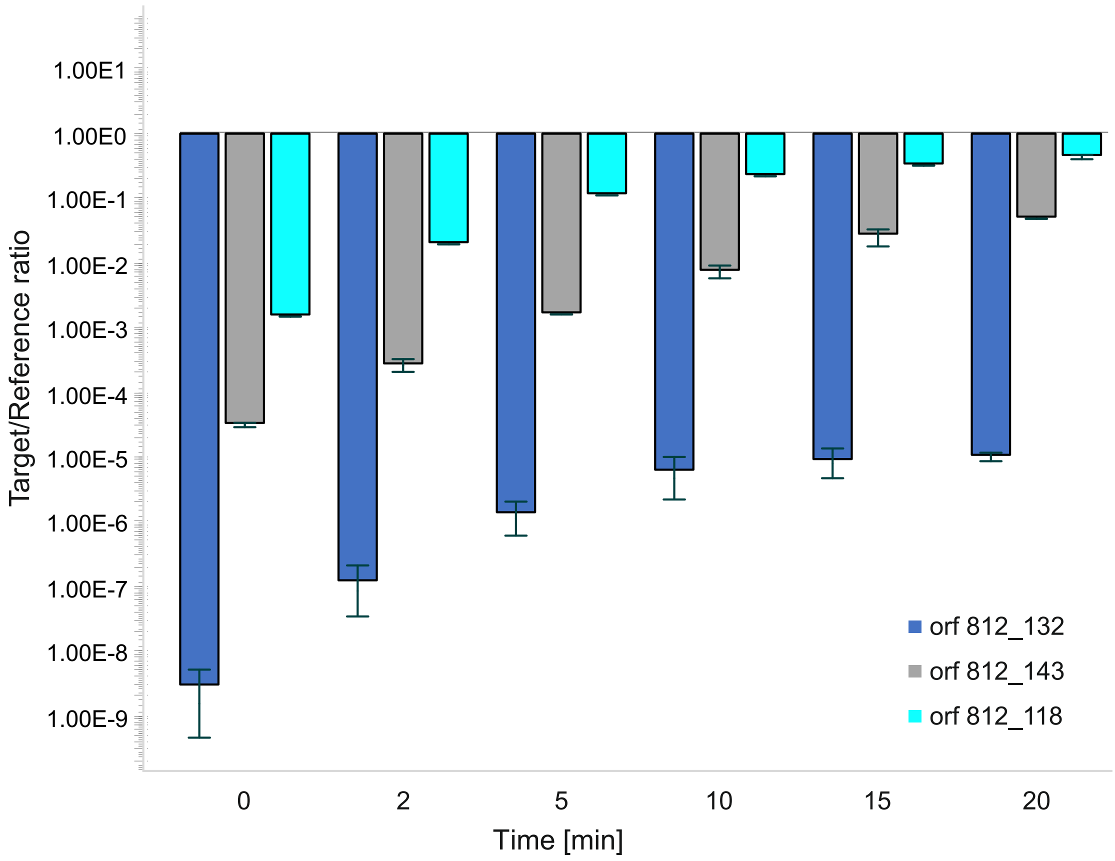

FIG S3 Relative quantification of phage 812 transcripts in *Staphylococcus aureus* RN4220 (53^{+}) using RT- qPCR. The Target/Reference ratio of the early- (anti-sigma factor, orf 812_132, AZB49840.1), middle- (putative DNA-binding protein, orf 812_143, AZB49851.1), and late-phase gene (baseplate wedge protein, orf 812_118, AZB49826.1) transcripts was calculated against bacterial reference gene *gyrA* for the DNA gyrase subunit A (Gene ID: 3919179) at different time points during phage replication.

The efficiencies of reaction (E) for each gene were calculated using the equation: $E = 10^{-1/slope}$; *gyrA*: E = 1.968; the slope -3.402, and error of the standard curve 0.0186; orf 812_132: E = 1.877; the slope -3.657, and error of the standard curve 0.103; orf 812_143: E = 1.930; the slope -3.502, and error of the standard curve 0.0115; orf 812_118: E = 1.908; the slope -3.563, and error of the standard curve 0.0090. Values in the logarithmic bar chart are in reverse order.
