# Supplementary material for: Staphylococcus aureus Prophage-Encoded Protein Causes Abortive Infection and Provides Population Immunity against Kayviruses
Source: mBio. 2023 Feb 13;14(2):e02490-22. doi: 10.1128/mbio.02490-22 (PMC10127798; doi:10.1128/mbio.02490-22)
Supplement: FIG S4 [file mbio.02490-22-s0008.pdf]

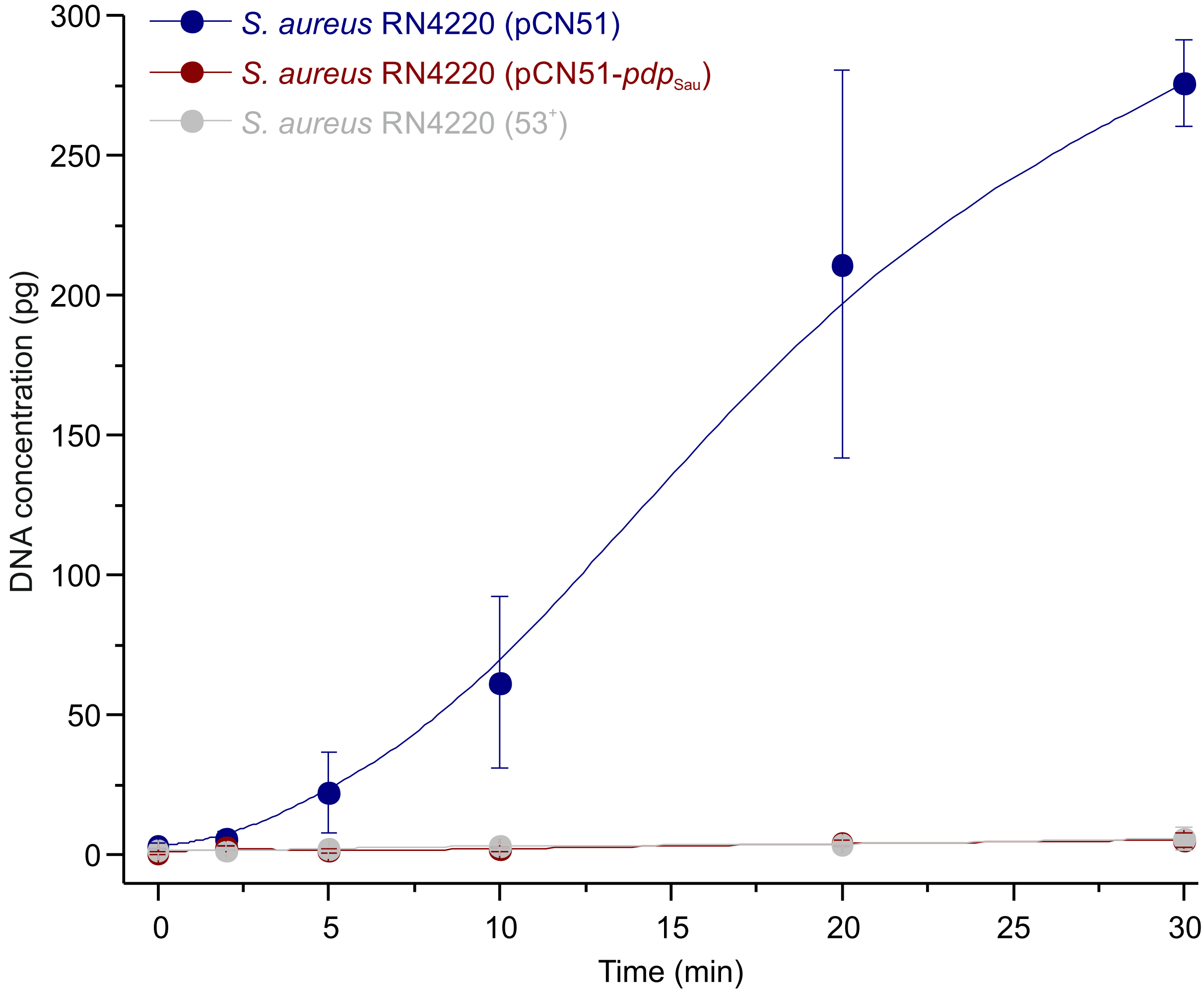

FIG S4 Absolute quantification of major capsid gene (*mcp*) using qPCR to indicate that phage DNA replication has stopped during 812 phage infection in two *Staphylococcus aureus* strains expressing the pdp_{Sau} compared to *S. aureus* RN4220 (pCN51). The efficiency of reaction (E) was calculated using the equation: $E = 10^{-1/slope}$, E = 1.816, the slope -3.860, and the error of the standard curve 0.0267.
